# The Added Value of Patient Engagement in Early Dialogue at EMA: Scientific Advice as a Case Study

**DOI:** 10.3389/fmed.2021.811855

**Published:** 2022-01-20

**Authors:** Aisling Murphy, Nathalie Bere, Spiros Vamvakas, Maria Mavris

**Affiliations:** ^1^European Medicines Agency, Amsterdam, Netherlands; ^2^GlaxoSmithKline, London, United Kingdom

**Keywords:** scientific advice (SA), regulation, medicines, patient engagement (PE), added value

## Abstract

The European Medicines Agency provides Scientific Advice to medicines developers and patient input has been an integral part of this process for many years. As end users of medicines, patients bring their perspectives to many different processes along EMA's regulatory pathway, complementing the scientific expertise. While the value of including patients has been well-demonstrated over the years, requests for evidence of their impact continue. Using Scientific Advice as a case study, data was collected over a four-year period to assess the number of patients involved, where they contributed, as well as the impact and added value of their input. In this paper, we show that patients' contributions have a tangible impact on the recommendations provided to developers and in over half of the cases, this led to further discussion on relevant patient perspectives. These data provide quantitative evidence of the value of patient input in medicines development and supports EMA's continued inclusion of their voice throughout the medicine's lifecycle.

## Introduction

Scientific advice is an important tool in the medicine regulatory lifecycle (1, 2). The European Medicines Agency (EMA) began offering scientific advice in 1996 to provide guidance to medicine developers on all aspects of the development programme from quality of the manufacturing process, to non-clinical and clinical aspects including methodological issues. The Scientific Advice Working Party (SAWP) makes recommendations in response to questions posed by medicines developers. Scientific advice aims to support developers to provide robust evidence for benefit–risk assessment at the time of marketing authorisation application (MAA), thereby facilitating the introduction of new, safe and effective medicines (3). While scientific advice is voluntary and non-binding, compliance with the recommendations has been shown to correlate with successful MAAs (4). Scientific advice is one of the earliest activities where EMA began engagement with patients.

Patients, as end users of medicines are key stakeholders of the Agency and are invited to contribute to EMA's work based on their experience of living with a particular condition and its treatment. The importance of involving patients in all aspects of medicines development is no longer disputed, yet questions concerning how best to capture and use their input and how to measure their impact are still being raised.

EMA has been actively engaging with patients since its creation in 1995, beginning with informal discussions with patient groups that have now evolved to more formalised interactions as set out in the Framework for interaction with patients and their organisations (5, 6).

Patient engagement has evolved and diversified over the years in parallel with the expansion of EMA's remit. There are several categories of patient representation at EMA; those who represent all patients in the European community as members of the EMA Management Board and scientific committees; those who represent their organisation via membership of EMA's Patients' and Consumers' Working Party (PCWP) or participation in workshops and responding to EMA public consultations. Finally, patients represent themselves as individual experts for medicine-related activities such as scientific advice, scientific advisory groups (SAGs) and the review of documents destined for the public such as medicines overviews, safety communications and package leaflets (7). Various engagement methodologies have been tested and implemented over the years, resulting in established procedures to include the patient voice all along the medicines regulatory lifecycle at EMA (8).

**Figure 1:**
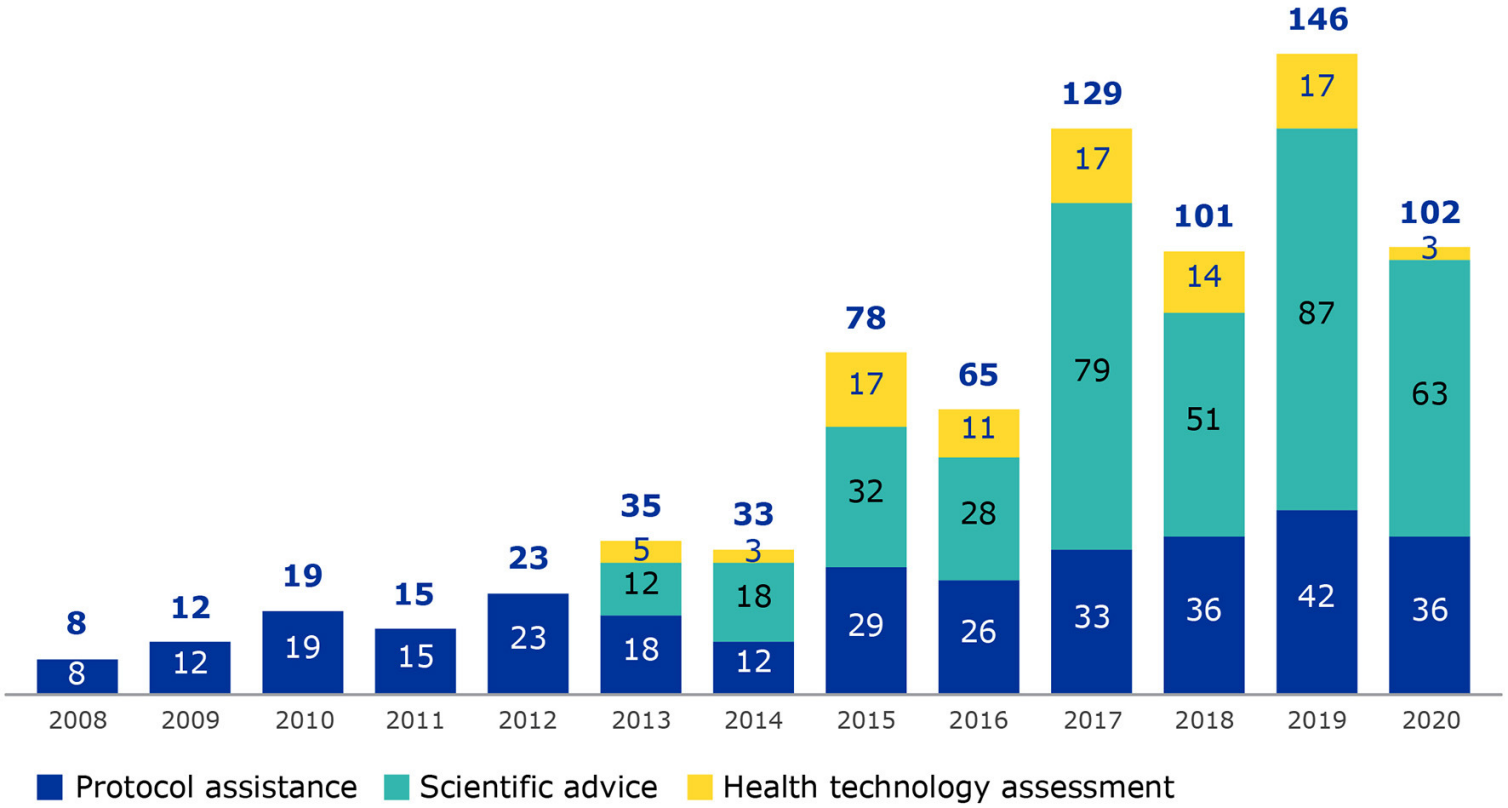
Number of patients involved from 2008 to 2020, by procedure type (protocol assistance, scientific advice and parallel procedures with HTA bodies). Data collection on patient involvement in scientific advice procedures began in 2008.

Patient involvement in scientific advice began in 2005 when rare disease patients requested to be involved in protocol assistance procedures for medicines with an orphan designation. Success of this activity led to the inclusion of patients in scientific advice for medicines without an orphan designation from 2013 as well as parallel procedures of scientific advice and health technology assessment (HTA) bodies (Figure 1). The term “scientific advice procedures” will be used to encompass scientific advice, protocol assistance (applicable to orphan medicines only) and parallel EMA/HTA bodies consultations (9).

Scientific advice was selected as EMA has many years of experience engaging with patients in this area, there is a good data set covering several years that represents a collaborative activity within EMA as well as with patient groups. A steady increase of patient involvement in scientific advice procedures has been observed over the years correlating with the increase in requests for scientific advice to EMA (8) and the increased efforts made by EMA for patient involvement. In this study we show the added value of patients' contributions to medicines development as well as to a broader understanding of living with the condition.

## Methodology

### Identification of Scientific Advice Procedures for Patient Input

Scientific advice requests that would benefit from patient involvement were identified during monthly meetings with EMA scientific advice office. Not all procedures required patient participation if, for example, advice is only sought on non-clinical, regulatory or statistical issues. Individual patients were invited primarily to comment on clinical aspects, such as comparator treatments, endpoints and patient populations in prospective clinical studies as these relate to the objectives and feasibility of the clinical studies.

### Identification of Patients

EMA works with a diverse group of EU patient organisations that meet strict eligibility criteria with respect to representation, funding and transparency (10). The term patient is used to encompass patients, consumers or carers. Patients may be identified and contacted through an EMA eligible patient organisation or via an EMA database of individuals, established in 2016, who wish to participate in EMA's activities. Patients who have registered their interest in participating may be contacted directly when a procedure in their disease area of interest arises. Currently more than 180 organisations and 500 individuals are registered in EMA's stakeholder database.

### Criteria for Patient Involvement

There are several criteria that were used to select patients; usually one and sometimes two patients are invited to participate in a scientific advice procedure. English is the working language at EMA, and all patients must have a level of understanding that would enable them to read the relevant documents and comment in writing or in person. Depending on the questions raised, the level of experience can vary from a newly diagnosed individual, a carer or a long-term patient advocate representing the condition. Having followed a training course on medicines' development is beneficial but not a pre-requisite for involvement. As with all other experts participating in EMA activities, patients were required to complete a confidentiality agreement and declare any competing interests, which were assessed prior to formal invitation. EMA experts were generally residents of an EU Member State.

### Collection of Feedback and Analysis of Patient Input

The EUSurvey tool (European Commission's official survey management) was used to create a survey and collect data related to patient involvement. The survey was created with colleagues in the EMA scientific advice team and contained 11 questions (Annex I).

The first part of the survey asks about the coordinators' perspective, whether they had any interactions, in writing or by telephone, with the patient prior to their participation in the procedure. This also included whether the patient was adequately prepared (with respect to their role and understanding of the procedure) as well as the areas where patient input was sought. Terminology used in the survey was consistent with terms used and understood by all coordinators. EMA colleagues responsible for specific scientific advice requests, referred to as procedure coordinators, were sent a survey at completion of each procedure (Day 40 for written procedures or Day 70 for procedures where a meeting was held).

Patients were also sent a survey (created in EUSurvey) at completion of each procedure to gather their perspectives on their involvement in the scientific advice procedure (Annex II). The survey was sent to patients at completion of each procedure along with a letter of thanks for their participation, meeting minutes (in the case a meeting was held) and the final letter of advice sent to the medicine developer. No personal data was collected via the surveys. Questions to the patients included whether what was expected of them was clear, if they had enough opportunity to contribute to the procedure and if they felt their comments were considered during the activity. As responding to the survey is voluntary, EMA did not follow up to obtain feedback unlike with the surveys completed by coordinators.

### Data Analysis

**Figure 2:**
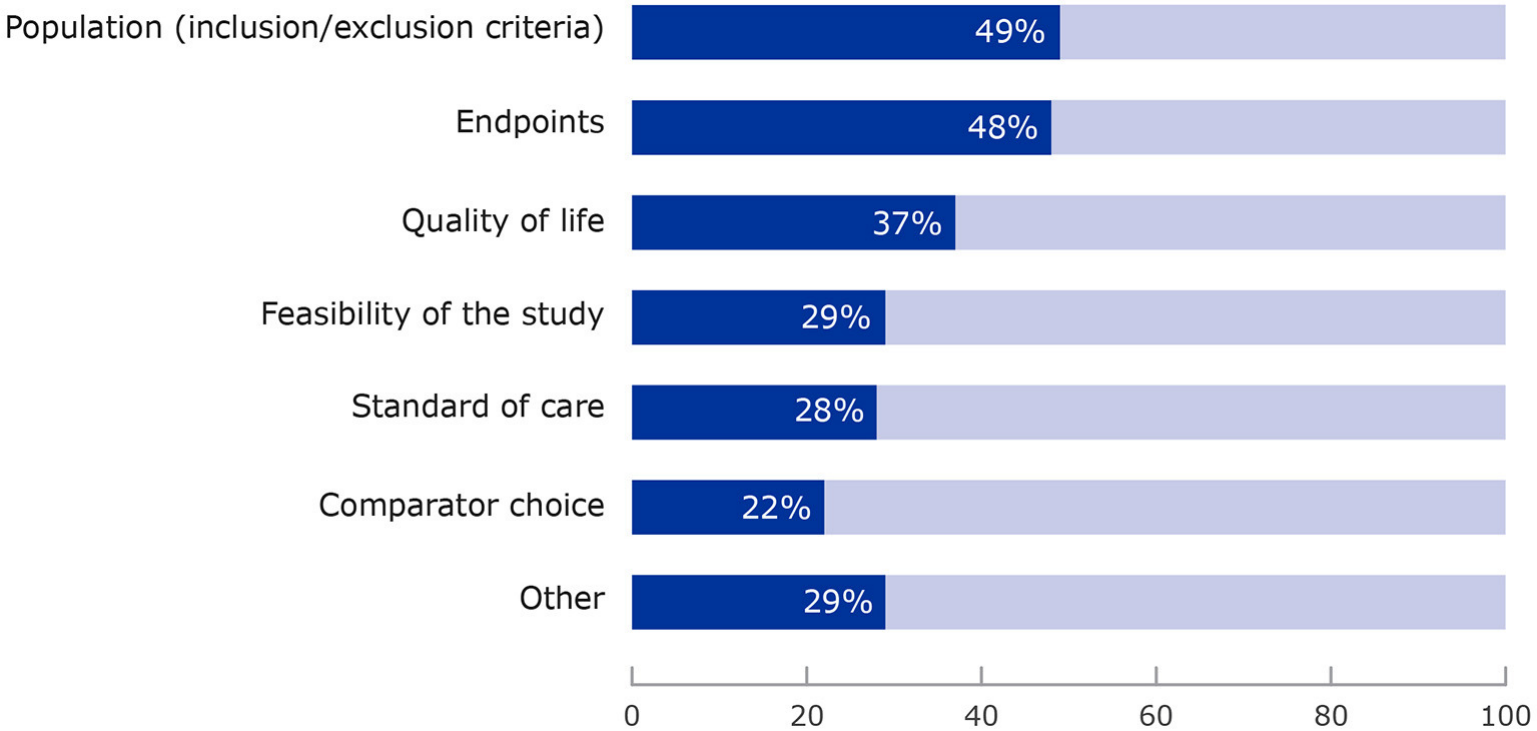
Areas of development plan where patients provided input. More than one category could be selected for each survey question.

**Figure 3:**
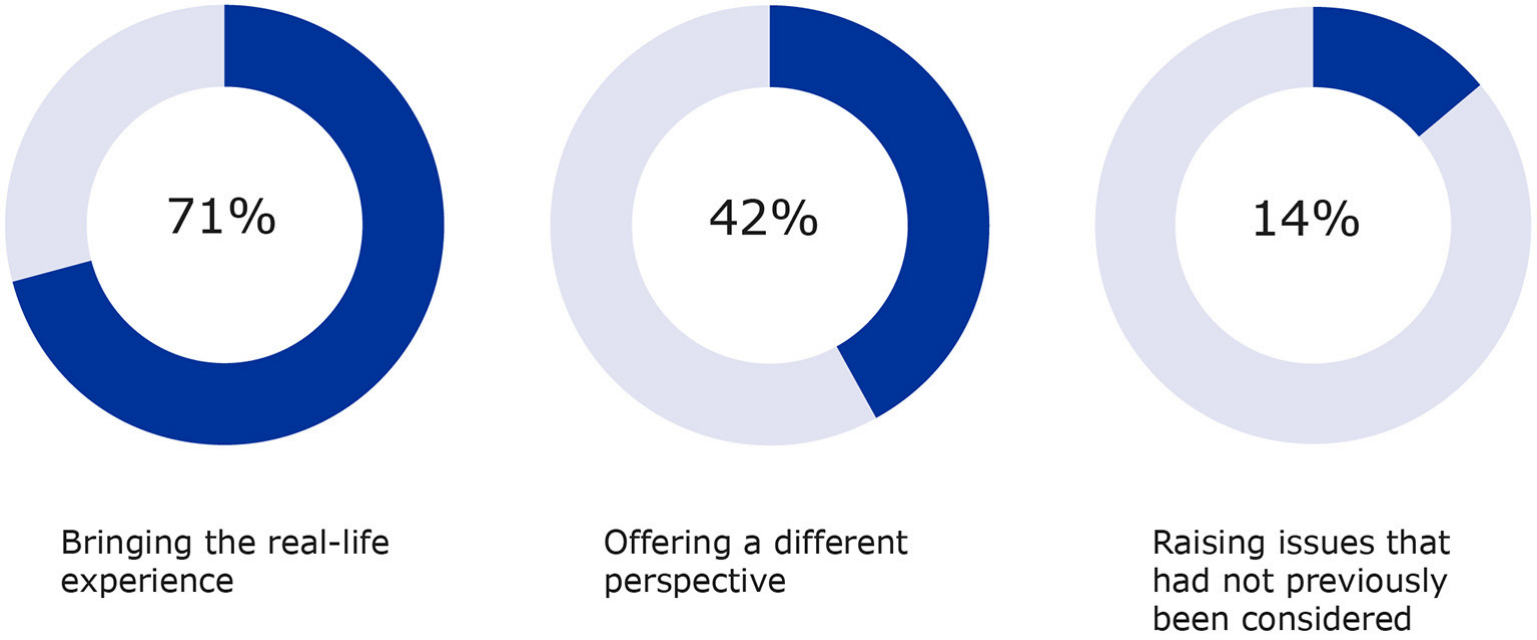
Additional input by patients on aspects such as real-life experience, different perspectives and other considerations were also measured (2017–2020).

A total of 371 survey responses were received from the procedure coordinators for the four-year study period. Analyses were performed using aggregated data for each survey question. To determine the percentages shown in Figures 2, 3, the total number of responses received for each question was divided by the total number of survey responses received (*n* = 371) to ensure a consistent denominator. Several survey questions allowed more than one response.

## Results

### Responses to Surveys by EMA Procedure Coordinators

For the study period of 2017–2020, a total of 371 survey responses were received for the 478 patients (78%) who were involved in scientific advice procedures related to clinical development (11). The results for each year, 2017 (90/129; 70%), 2018 (75/101; 74%), 2019 (110/139; 79%) and 2020 (96/102; 94%), show high response rates. On average patients are involved in one in five (20%) scientific advice procedures that include clinical questions (8).

#### Contact With the Patients

The majority of coordinators (91%) contacted the patients prior to their involvement to explain the process of scientific advice and where their input would be helpful.

#### Areas for Patient Input

Requests by coordinators focused primarily on aspects such as study population (77%), endpoints (74%), study feasibility (52%), quality of life (48%) and other aspects such as patient-reported outcomes, biomarkers and safety issues.

#### Where Patients Made Contributions

Figure 2 shows that patients most often commented on the selection of the clinical trial population (49%), the choice of endpoints (48%), study feasibility (52%), quality of life studies (48%), and also bringing in a real-life perspective of living with a condition (as patient or carer), offering a perspective different to the medical and scientific experts and raising issues that had not previously been considered by the Scientific Advice Working Party. “Other” areas included general insights into the condition, its daily impact and treatment options. Overall, input resulted in further reflection by the working party in more than half of the cases (52%).

The survey also measured whether the recommendations provided to the developer were modified as a result of patient input. The results showed that the final advice letter was modified in 20% of cases based on patient contributions. Importantly, the vast majority of cases where patient input did not change the final advice, is correlated to the fact that patients agreed with the proposed development plan.

The added value of patient input was measured for the areas listed in Figure 3 with “bringing the real-life experience of living with a condition and its treatment” ranking highest (71%), followed by “offering a different perspective” being outlined (42%) as well as “raising issues that had not previously been considered” (15%). These aspects complement the contributions to the specific questions raised by the developers on the clinical trial aspects and contribute to future recommendations in the same therapeutic area.

### Responses to Surveys From Patients

EMA also received 125 survey responses from participating patients for the same reporting period. Participants could contribute to a scientific advice procedure in writing or in person when a meeting with the medicine's developer was organised.

Participation in scientific advice: almost equally split between contributing in writing (49%) or attending a meeting (51%). There was some overlap as some patients who attended meetings also provided comments in writing.

In most cases (86%), patients responded that they understood what was expected of them in terms of their written contribution and 83% felt that they were able to provide input to the issues raised in the scientific advice request.

Patients who attended meetings (in person or virtually) reported in 90% of cases that they understood what was expected of them in the meeting and felt in 92% of the cases that they had an opportunity to provide input to the discussion.

Overall 75% of patients felt their comments were taken into account, both in writing and while attending meetings but when looking at the breakdown, there is a higher response rate (86%) when patients attended meetings compared with 76% when contribution was only sought in writing.

The majority of patients (80%) felt positive about their overall experience of participation. The main barriers identified by patients were the complexity of the information to review and the short deadlines for contributing particularly during written procedures.

## Discussion

In this paper we describe the contributions and added value of patient participation in scientific advice procedures at EMA, which has not previously been assessed in a quantifiable manner. We describe the methodology used to involve patients in scientific advice and present an analysis of feedback received from the EMA procedure coordinators as well as the patients who have participated.

While regulators and other experts can provide guidance on many aspects of the complexities of medicine development, the day to day experience of living with a condition and its treatment can only be addressed by someone with first-hand experience. The data presented here offers unique insights as it is the first time that such impact data is being presented by a regulatory body. We have highlighted how patients fill an important gap by providing real-life experience of the conditions and their treatments, in addition to providing input into the clinical aspects of the development plans.

As a result of patient input, one in five scientific advice responses provided to the medicines' developers were modified and in 90% of the cases where no modification was made, patients agreed with the proposed development plan. Overall, their contributions led to additional reflection by the EMA procedure coordinators in more than half of the cases. This demonstrates that there are two levels of impact which can be considered; first where patients input results in a change to the recommendations provided by a medicines' regulator to a developer, second where patients agree with a proposed development plan, therefore not necessitating additional changes to the advice given. In addition, patients who were contacted prior to the procedure starting appeared to be better prepared than those who were not.

Our analysis supports the continued involvement of patients in scientific advice and illustrates the importance of including this stakeholder group in early dialogue between regulators and medicines' developers. There is clear alignment of both EMA procedure coordinators and patient participants that patient involvement in this activity is beneficial. In nearly all cases, EMA procedure coordinators indicated that patient participation was of added value and the majority of patients felt that their comments were impactful.

The authors acknowledge that further analysis could be performed on survey responses per therapeutic area of conditions for which scientific advice was sought. Another limitation to acknowledge is that the patients involved in the procedures across the years were not always the same and a diversity of experience and input would be observed due to the mix of those who were new to EMA activities and those more experienced patient experts. While the questions related to the different aspects of the development plan are clear well-defined for the scientific coordinators, questions related to the additional value brought by patients such as “bringing the real-life experience”, “offering a different perspective” and “raising issues not previously considered” could be considered subjective and thus open to interpretation by individual coordinators.

The feedback from patients is also encouraging. One respondent described their involvement as “a highlight in 17 years of patient advocacy work” and another commented “I really appreciate the relevance EMA gives to patients” voice in the procedures. Taking into consideration our opinion from the beginning, it is beneficial for all stakeholders'.

The complexity of the information on which patient input is sought and the regulatory timelines of scientific advice were difficulties raised by some respondents. EMA aims to lessen this as much as possible by asking patients to focus on the sponsor's clinical questions and by providing one-to-one individual support throughout the procedure. Importantly, our analysis shows that patients were more likely to be more prepared to participate when they had been contacted by the EMA procedure coordinator prior to their involvement. The importance of prior contact is crucial as it allows for better preparation and thus more meaningful contributions by patients. In addition to one-to-one support provided by EMA staff, patients participating in EMA activities can benefit from various multimedia training resources online (12). EMA also holds stakeholder training days where attendees participate in interactive small group sessions on various regulatory activities including scientific advice.

We acknowledge that the involvement of only one or in some cases two patients per procedure can mean that the views expressed are not necessarily representative of the entire patient community in a given disease area. EMA is exploring additional methods to gather input from the wider patient community. Following the publication of a patient preference study involving multiple myeloma patients in 2016 (13), EMA is exploring the feasibility of conducting similar studies in other disease areas. EMA also collaborates with the IMI-PREFER project, a consortium of stakeholders who have explored the use of patient preference studies in regulatory, academic and industry settings (14). In addition, the Agency is examining the possibility of facilitating focus groups to gather the opinions of several patients on a given topic. The use of focus groups and patient preference elicitation will complement rather than replace one-to-one discussions involving individual patients. Each methodology has value and addresses different needs. Together these activities will help to further develop and strengthen the patient voice in regulatory procedures, which is further reinforced by the recommendations in the EMA Regulatory Science to 2025 (15) and comments received during the public consultation (16).

It is important to bear in mind that patients contributing at European level can also provide their expertise at national level. We hope our findings encourage national competent authorities who have not yet involved the patient voice in their procedures to explore this possibility.

## Conclusions

Our analysis illustrates how patient input enriches and complements the medical and scientific discussions in EMA scientific advice procedures. Patients provide their perspectives on a wide spectrum of clinical questions posed by medicines' developers. Patient input adds value in many ways as they offer a different perspective to other experts; they bring experience of living with the condition and its treatment into the discussion. They raised issues that had not been previously considered and, in some cases, they agreed that regulators and developers are taking the right steps. Impact is not only measured by making changes or disagreeing with the recommendations. Importantly we have demonstrated that patients' contributions to these procedures make a difference and that their suggestions lead to concrete additions to the final scientific advice issued.

The added value of patient input is not exclusive to scientific advice procedures and they are involved in other regulatory procedures such as scientific advisory groups and in consultations by EMA committees, which are both systematic and evolving at EMA. Thus, the demonstrated value of patient inclusion in scientific advice not only supports EMA's continued inclusion of the patient voice throughout the medicine's lifecycle and the diversification of activities where patients participate, but also provides tangible evidence of impactful importance of engaging with patients. There is a need to further expand patient input to real-world evidence, patient reported outcomes, patient preferences and patient experience data, which can only be to the benefit of public health in the EU.

## Data Availability Statement

The datasets presented in this article are not readily available because the data contain commercial confidential information as well as identifiable patient data. Analysed data can be shared but not raw data. This research did not require ethics approval or informed consent. Requests to access the datasets should be directed to maria.mavris@ema.europa.eu.

## Ethics Statement

Ethical review and approval was not required for the study on human participants in accordance with the local legislation and institutional requirements. Written informed consent from the participants was not required to participate in this study in accordance with the European Legislation and the Institutional Requirements.

## Author Contributions

All authors listed have made a substantial, direct, and intellectual contribution to the work and approved it for publication.

## Conflict of Interest

AM conducted the first year of research during her traineeship at EMA and subsequently was employed with GlaxoSmithKline, United Kingdom. The remaining authors declare that the research was conducted in the absence of any commercial or financial relationships that could be construed as a potential conflict of interest.

## Publisher's Note

All claims expressed in this article are solely those of the authors and do not necessarily represent those of their affiliated organizations, or those of the publisher, the editors and the reviewers. Any product that may be evaluated in this article, or claim that may be made by its manufacturer, is not guaranteed or endorsed by the publisher.
