# Supplementary material for: The Added Value of Patient Engagement in Early Dialogue at EMA: Scientific Advice as a Case Study
Source: Front Med (Lausanne). 2022 Jan 20;8:811855. doi: 10.3389/fmed.2021.811855 (PMC8811124; doi:10.3389/fmed.2021.811855)
Supplement: Supplementary file 1 [file Data_Sheet_1.PDF]

PM feedback on Patient involvement in SA/PA

Fields marked with * are mandatory.

Product name

Product Manager (Scientific Officer) name:

* 1. Product name

* 2. Did the patient seem adequately prepared for the procedure (i.e. demonstrate a general understanding of scientific advice and the procedure under discussion)?

- ○ Yes
- ○ No
- ○ Difficult to assess

* 3. Did you interact with the patient prior to their involvement?

- ☐ Yes, by telephone
- ☐ Yes, by email
- ☐ No

If no, please explain

* 4. What particular issues in the procedure did you highlight to the patient?

- ☐ Population (inclusion/exclusion criteria)
- ☐ Feasibility of the study
- ☐ Endpoints
- ☐ Comparator choice
- ☐ Quality of life
- ☐ Standard of care
- ☐ Other

If other, please specify

* 5. How did the patient participate in a SA/PA procedure?

- In writing - Joint Report
- In person - Discussion Meeting

Patient input

* 6. What was the level of the patient`s activity/comments/contribution on the development plan?

- Substantial comments
- Some comments
- Agreed with proposed responses
- No contribution

* 7. Did the patient agree with the CHMP responses in the scientific advice request?

- Yes, overall
- Yes, for the majority
- Some disagreements

If there was disagreement, please explain

* 8. Did any of the patient`s comments result in further reflection by the coordinators?

- Yes
- No

* 9. Did any of the patient`s input result in a modification of the final advice letter?

- Yes
- No

Please, explain your choice

* 10. In which aspect of the development plan did the patient make input?

- [ ] Population (inclusion/exclusion criteria)
- [ ] Feasibility of the study
- [ ] Endpoints
- [ ] Comparator choice
- [ ] Quality of life
- [ ] Standard of care
- [ ] Other

If other, please specify

* 11. What was the added value of the patient`s input?

- [ ] Raising issues that had not previously been considered
- [ ] Offering a different perspective
- [ ] Bringing the real-life experience
- [ ] None
- [ ] Other

If other, please describe how their input was beneficial as this information is included in the annual report to the EMA Management Board
