# Supplementary material for: The Added Value of Patient Engagement in Early Dialogue at EMA: Scientific Advice as a Case Study
Source: Front Med (Lausanne). 2022 Jan 20;8:811855. doi: 10.3389/fmed.2021.811855 (PMC8811124; doi:10.3389/fmed.2021.811855)
Supplement: Supplementary file 2 [file Data_Sheet_2.PDF]

Patient Participation in Scientific Advice/Protocol Assistance

Fields marked with * are mandatory.

1. Product Name

* 1. Product name and date of consultation:

2. Pre-meeting

* 1. I received sufficient and understandable background information regarding scientific advice/protocol assistance in general:

- 1 (Agree)
- 2
- 3
- 4
- 5 (Disagree)

* 2. I received sufficient and understandable information on this specific consultation:

- 1 (Agree)
- 2
- 3
- 4
- 5 (Disagree)

3. Participation

* 1. Did you attend a discussion meeting at EMA or contribute in writing?

- Attended discussion meeting
- Contributed in writing
- Neither

4. Contribution in Writing

* 1. I understood what was expected of me in terms of my contribution in writing:

- 1 (Agree)
- 2
- 3
- 4
- 5 (Disagree)

* 2. I was able to provide input on the issues under discussion:

- 1 (Agree)
- 2
- 3
- 4
- 5 (Disagree)

5. Discussion Meeting

* 1. The meeting arrangements were well taken care of (travel, access to EMA, etc..):

- 1 (Agree)
- 2
- 3
- 4
- 5 (Disagree)

* 2. I understood what was expected for my participation at the meeting:

- 1 (Agree)
- 2
- 3
- 4
- 5 (Disagree)

* 3. I was given adequate opportunity to provide input to the discussion:

- 1 (Agree)
- 2
- 3
- 4
- 5 (Disagree)

6. Feedback

* 1. I feel my comments were taken into account:

- 1 (Agree)
- 2
- 3

- 4
- 5 (Disagree)

* 2. How do you feel overall about your participation in this activity?

- 1 (Positive)
- 2
- 3
- 4
- 5 (Negative)

3. If you did not contribute to this particular consultation, could you tell us why?

4. Any additional comments?
